# Cooperative behavior in adolescents: a contribution of empathy and emotional regulation?

**DOI:** 10.3389/fpsyg.2024.1342458

**Published:** 2024-04-04

**Authors:** Eduardo Salvador Martínez-Velázquez, Sandra Pamela Ponce-Juárez, Alfonso Díaz Furlong, Henrique Sequeira

**Affiliations:** ^1^Laboratory of Psychophysiology, Faculty of Psychology, Benemérita Universidad Autónoma de Puebla, Puebla, Mexico; ^2^Admissions Department and Academic Follow-Up, Vicerectory of Teaching, Benemérita Universidad Autónoma de Puebla, Puebla, Mexico; ^3^Laboratoire de Sciences Cognitives et Affectives (SCALab), CHU Lille, UMR CNRS 9193 – University of Lille, Lille, France

**Keywords:** cooperative behavior, empathy, emotional regulation, development, adolescent

## Abstract

**Aim:**

This study aims to identify different levels of empathy and emotional regulation along adolescent years and their relationship with cooperative behavior.

**Methods:**

Eighty healthy males were divided into four age groups: 20 Early Adolescents, 20 Middle Adolescents, 20 Late Adolescents and 20 Adults. Participants responded to empathic and emotional regulation scales, then were assigned to an unknown partner to perform the prisoner’s dilemma paradigm.

**Results:**

The statistical analyses allowed to distinguish the groups on the basis of the components making up the two scales: scores on the Perspective Taking component were higher for Adults and Late Adolescents participants than for Middle Adolescents and Early Adolescents groups (*p* < 0.05); scores on the Personal Distress component were higher for Early Adolescents group than for Late Adolescents and Middle Adolescents groups (*p* < 0.05); scores on the Difficulties engaging in goal directed behavior component were higher for Middle Adolescents and Early Adolescents groups than for Adults group (*p* < 0.05). We observed differences between groups (*p* < 0.001) with higher number of cooperation responses in Adults compared to Middle Adolescents (*p* < 0.05) and Early Adolescents groups (*p* < 0.001).

**Discussion:**

These findings suggest that the cooperative behavior changes during the different stages of adolescence seem to be related to the development of empathy and emotional regulation components.

## 1 Introduction

Cooperative behavior involves the interaction of two or more people who work together towards a common goal, with mutual benefits for everyone (Gutiérrez-Roig et al., 2014; Nava et al., 2023). Some studies have suggested that the tendency to show cooperative behaviors could change with age (Garaigordobil and García de Galdeano, 2006; Gilar Corbi et al., 2008; Eisenberg et al., 2010; Belli et al., 2012; Smith et al., 2013; Gutiérrez-Roig et al., 2014; Keil et al., 2017; Taheri et al., 2018; Castellano Navarro et al., 2019; Zhang et al., 2019; Nava et al., 2023). In this direction, adolescents show a higher tendency to cooperate than children (Fuentes and Fernández, 1993; Gutiérrez-Roig et al., 2014; Keil et al., 2017; Castellano Navarro et al., 2019), while adults show more cooperative behaviors than adolescents (Belli et al., 2012; Taheri et al., 2018; Zhang et al., 2019; Nava et al., 2023).

Several factors could influence cooperative behavior, and they had been explored through various types of paradigms such as the prisoner’s dilemma or trust games. In a study with adults (19–35 years old) and adolescents (13–14 years old) who participated in a trust game, adults showed prosocial behaviors and skills to reduce conflicts whereas adolescents presented rather equitable behaviors (Belli et al., 2012). The authors noted, however, that these results could be influenced by perceived proximity of their playmate, for example, being in an adjacent room. This perception of the proximity of the partner (Belli et al., 2012) and other factors like the environmental context (Taheri et al., 2018), the virtual or in-person situation (Miwa and Terai, 2012; Taheri et al., 2018), a reciprocal behavioral (Gutiérrez-Roig et al., 2014; Nava et al., 2023) and the belief of cooperation (Zhang et al., 2019) shown to influence cooperative behavior in adolescents and adults. Nonetheless, particularly during adolescence, the results have been inconsistent about the cooperative behaviors: some studies being in favor of these behaviors (Gutiérrez-Roig et al., 2014; Keil et al., 2017) while others supported equitable (Belli et al., 2012) or even non-cooperatives behaviors (Nava et al., 2023).

The period of adolescence leads to major biological, psychological and social changes which evolve through three main stages and having links with the development of self-regulation (Gaete, 2015; Tamayo Lopera et al., 2018). Early adolescence (10–14 years old) is characterized by egocentrism, a deficit in impulse control, and the search for immediate gratification. During this stage, adolescents assume that others have the same perspectives and values as themselves which lead to the emergence of empathic behaviors (Gaete, 2015). In middle adolescence (15–17 years old) occur important changes in brain structures driving new processes (judgment, decision, self-control) able to modulate emotional behaviors. During this period, individuals acquire the ability to observe the feelings of others and feel worried about them. In addition, they have a self-image that is highly dependent on the opinion of third parties (Gaete, 2015). Prior studies support these processes by showing that early adolescents tend to develop behaviors predominantly influenced by the activity of the amygdala while adolescents from 17 years old show patterns more similar to adults (planning, reasoning, impulse control and emotional regulation), known to be mainly controlled by the activity of the frontal lobes (Papalia et al., 2012; Gaete, 2015; Tamayo Lopera et al., 2018). Finally, in late adolescence (18–21 years old), people show increased ability to predict consequences, solve problems, and make decisions on their own (Gaete, 2015).

Some authors (Garaigordobil and García de Galdeano, 2006; Gilar Corbi et al., 2008; Eisenberg et al., 2010; Smith et al., 2013; Keil et al., 2017; Castellano Navarro et al., 2019) proposed that, during adolescence, cooperative behaviors could also be influenced by individual characteristics, such as cognitive and emotional skills, largely dependent on brain development. In this frame, some authors pointed out the role of self-regulation as a central disposition to prosocial behavior and to the inhibition of aggressive behavior (Eisenberg et al., 1994; Eisenberg, 2000; Mestre Escrivá et al., 2002). In particular, individuals with high emotional regulation are more likely to experience perspective-taking empathy rather than personal distress (Mestre Escrivá et al., 2002). Emotional regulation implies monitoring, evaluating, and modification of emotional reactions, especially the power to control the intensity and temporality to achieve goals (Hervás and Jódar, 2008; Stifter and Augustine, 2019). In a complementary way, empathy is the ability to identify and understand the thoughts (cognitive empathy) and moods (affective empathy) of another person (Davis, 1980; Fernández Pinto et al., 2008). Therefore, the development of emotional regulation and empathy resources, associated with brain maturity along the adolescent period could predispose to increase cooperative behaviors (Eisenberg, 2000; Balconi et al., 2020). However, the changes in both factors and their potential links with cooperative behavior during the different stages of adolescence remain to be explored. Moreover, most studies exploring cooperative behavior in adolescents have worked with samples without having distinguished the different stages typical of the adolescent period, which involves important cognitive and emotional changes (Gaete, 2015; Tamayo Lopera et al., 2018). In this frame, the inconsistences about the cooperative behavior of adolescents (Belli et al., 2012; Gutiérrez-Roig et al., 2014; Keil et al., 2017; Nava et al., 2023) could be explained by variations associated to successive levels of the adolescence evolution. Thus, it is still unknown whether the emotional regulation or empathy skills during the different stages of adolescence (early, middle, and late adolescence) are linked or not to the cooperative behavior as observed in adulthood (Eisenberg et al., 2010; Glazer, 2021). The ability to identify factors that interact with cooperative behavior could help to develop educational models able to favor the development of cooperative behavior and its beneficial social consequences.

Hence, the aim of the present study was thus twofold: (1) to identify the levels of empathy and emotional regulation during the different stages of adolescence (early, middle, and late adolescence) and adulthood; (2) to assess the link between these levels and the development of a cooperative behavior. To this end, emotional regulation and empathic capacities of healthy individuals were assessed before to be invited to perform the prisoner’s dilemma paradigm, often used to explore cooperative behaviors.

We hypothesize the following: (1) the group of Early and Middle Adolescents will present lower scores than the group of Late Adolescents and Adults in cooperative responses to the dilemma of prisoners, and (2) we will observe a positive correlation between empathy, emotional regulation, and cooperative behavior in the four groups.

## 2 Materials and methods

All participants gave written informed consent following the international declaration of ethics of Helsinki. This study was registered and approved by the research and ethics committee of the Department of Psychology of the University (SIEP 140/2021). For minor age adolescents, the father, mother, or tutor signed the consent.

### 2.1 Participants

We distributed 80 healthy men into four groups according to their age: EA – 20 Early Adolescents (12–13 years old), MA – 20 Middle Adolescents (16–17 years old), LA – 20 Late Adolescents (19–20 years old), and AD – 20 Adults (23–24 years old). As an additional inclusion criterion for gender and age, we included an intelligence score corresponding to each age-based Barranquillas Rapid Test (BARSIT), which comprises 60 items (Del Olmo, 1980). The criteria were: a score ≥ 38 in the case of Early Adolescents and Middle Adolescents and a score **≥** 43 for Late Adolescents and Adults. We considered the intelligence score as an inclusion criterion because a score ≥ 38 to the BARSIT indicate that the participants have developed verbal reasoning and the capacity for abstraction and synthesis (Del Olmo, 1980), which we consider important to understand the task. In addition, we did not include participants with the following characteristics: history of neurological damage, addiction to toxic substances, and psychotic symptoms.

### 2.2 Empathy and emotional regulation questionnaires

We evaluated the components of empathy through the Interpersonal Reactivity Index (IRI; Davis, 1983; Ahuatzin González et al., 2019), which is one of the most widely used self-report questionnaires due to its multidimensional ability to approach empathy. The IRI comprises 28 items and includes four sub-scales: Perspective Taking (PT), Fantasy (FS), Personal Distress (PD), and Empathic Concern (EC). A suggested dichotomy of these components considered that the PT and FS sub-scales evaluate cognitive empathy while the other two sub-scales assess affective empathy (Fernández Pinto et al., 2008; Olivera et al., 2011; Guzmán González et al., 2014; Ahuatzin González et al., 2019). We applied the version of the IRI according to population (Ahuatzin González et al., 2019). We evaluated emotional regulation with the Difficulties in Emotional Regulation Scale (DERS; Marín Tejeda et al., 2012); the DERS comprises 36 items distributed into three six factors: Nonacceptance of emotional responses (NONACCEPTANCE), Difficulties engaging in goal-directed behavior (GOALS), Impulse control difficulties (IMPULSE), Lack of emotional awareness (AWARENESS), Limited access to emotion regulation strategies (STRATEGIES) and Lack of emotional clarity (CLARITY). For both questionnaires, the participants completed each item on a 5-point scale from ‘strongly disagree’ to ‘strongly agree’ (Marín Tejeda et al., 2012; Ahuatzin González et al., 2019).

### 2.3 Paradigm

The Prisoner’s Dilemma paradigm involves two players who must choose between two answers, cooperate or defect. In each trial, the players have only one opportunity to answer and have no information regarding their partner’s choice. We have our own version based on previous studies (Axelrod and Dion, 1988; Tejada et al., 2004; Zhang et al., 2019), in which each response combination provided different rewards: a/S (sucker), the worst possible reward and results when one of the players cooperates and the other defect; b/P (punishment), punishment for mutual desertion; c/ R (reward) is the reward for reciprocal cooperation; d/T (temptation), the highest possible reward and temptation to defect. The assignment of values was under the rule “S < P < R < T,” in which participants obtained the reward when one of the players deserted, meanwhile, the other one cooperates. This must always be higher, even more, than the situation in which both decide to cooperate. For more details, see Axelrod and Dion (1988) and Tejada et al. (2004).

We programmed a Prisoner’s Dilemma paradigm and we adapted the instructions to understand adolescents. We performed a pilot test with adolescents to corroborate it. The instructions were the following: “You and your partner, in a hypothetical situation, have been arrested because you committed a serious crime. However, there is no clear evidence against you, but there are strong indications of said crime and an offense. You and your partner will be interrogated, simultaneously in separate rooms, and each will be earning points to be released. The player with more points in favor will be released from both offenses, but his partner will be convicted. However, if the points in favor result in a tie, both will be acquitted of the crimes. During the interrogation, you will be able to know the result of your partner, and he will be able to know yours at the end of each question. Half of groups received the first answer as cooperation and the other half as defect.

### 2.4 Procedure

The procedure consisted of three main phases. Firstly, we recruited the participants through social networks based on the inclusion criteria of gender and age. Thereafter, we contacted them by video call in order to explain the procedure and provided them with the informed consent to be signed. For the minor age participants, we asked for authorization from the parent or tutor to perform all the activities. Secondly, we conducted a brief interview and applied the BARSIT, IRI sub-scales, and DERS factors questionnaires in a counterbalanced manner. In the third phase, we carried out a new session through video call. On this occasion, we assigned to each participant an unknown partner with who he would play the prisoner’s dilemma. The partner was an accomplice experimenter who helped us give the illusion of interacting in the paradigm. The partner only spoke to introduce himself and let us know that he understood the instructions. All participants had the same partner and name, and we placed a letter as a profile picture. Before beginning the paradigm, the instructions were to have their microphones on all time but the camera off; furthermore, we asked them not to talk during the game and to share their screen with the experimenter. Subsequently, we presented the instructions of the paradigm on the screen, and before starting the game, participants performed a test to get a better understanding.

### 2.5 Data analysis

We analyzed age, psychometric total scores and sub-scales factors to BARSIT, IRI, DERS, and the number of choices when the participants selected “cooperate” or “defect” on the paradigm, with a one-way ANOVA and *post hoc* Bonferroni tests. Further, we analyzed cooperation responses according to a two-way repeated-measures design by considering the groups (Early Adolescents, Middle Adolescents, Late Adolescents and Adults) and *post hoc* Bonferroni tests. Effect sizes have been calculated based on partial η^2^. The correlations between the variables were tested with the Pearson correlation coefficient (*r*).

## 3 Results

We were able to confirm the differences between groups concerning age, according to the results of ANOVA [*F*(3) = 1.7; *p* < 0.001, η^2^ = 0.98]. Furthermore, we differentiated groups by Intelligence based on the BARSIT score [*F*(3) = 9.9; *p* < 0.001, η^2^ = 0.28] as expected. These results can be considered as normal because the scores correspond to the expected level for each age (Del Olmo, 1980). The Adults group, particularly, showed higher scores than Middle Adolescents (*p* = 0.001), and Early Adolescents (*p* < 0.001), but not Late Adolescents (*p* = 1.000). Similarly, we did not find differences between the Middle Adolescents and Early Adolescents groups (*p* = 1.000).

### 3.1 Empathy and emotional regulation

According the differences between groups on empathy and emotional regulation scores, the Tables 1, 2 summarize the results. We did not find differences between groups in the total score of IRI [*F*(3) = 2.3; *p* = 0.084]. However, we could distinguish the groups by some sub-scales [*F*(3) = 7.6; *p* < 0.001, η^2^ = 0.23]. Perspective Taking sub-scale was higher for Adults and Late Adolescents participants than for Middle Adolescents and Early Adolescents groups (*p* < 0.05). Besides, the Personal Distress sub-scale distinguishes the Early Adolescents group with a higher score than Late Adolescents and Middle Adolescents (p < 0.05). Neither were observed differences between groups according to the total score of DERS [*F*(3) = 1.06; *p* = 0.370], but the Adults group showed a lower score than Middle Adolescents and Early Adolescents groups (*p* < 0.05) in the Difficulties engaging in goal-directed behavior sub-scale [*F*(3) = 3.35; *p* < 0.05, η^2^ = 0.02].

**Table 1:** Mean and standard deviation values for each sub-scale of the Interpersonal Reactivity Index (IRI) for the four groups.

	AD	(*n* = 20)	LA	(*n* = 20)	MA	(*n* = 20)	EA	(*n* = 20)	Difference	*M*	*SD*	*M*	*SD*	*M*	*SD*	*M*	*SD*	*p*-value
PT	26.55	4.77	24.20	3.53	22.45	4.13	20.55	4.01	AD, LA > MA*, AD > EA**
FS	22.40	4.37	21.20	4.64	21.40	5.57	18.75	5.31	ND
EC	25.75	5.02	23.90	4.19	23.75	4.24	22.40	4.24	ND
PD	21.70	4.62	20.40	4.47	21.25	4.85	25.15	3.39	EA > MA*, LA**

AD, adults; LA, late adolescents; MA, middle adolescents; EA, early adolescents; PT, perspective taking; FS, fantasy; EC, empathic concern; PD, personal distress; M, mean; SD, standard deviation. *p < 0.05; **p < 0.01; ND, no difference.

**Table 2:** Mean and standard deviation values for each factor of Difficulties Emotional Regulation Scale (DERS) for the four groups.

	AD	(*n* = 20)	LA	(*n* = 20)	MA	(*n* = 20)	EA	(*n* = 20)	Difference	*M*	*SD*	*M*	*SD*	*M*	*SD*	*M*	*SD*	*p*-value
NONACCEPTANCE	8.20	7.37	7.55	7.46	10.30	10.49	8.70	8.05	ND
GOALS	5.85	3.16	6.55	3.60	8.75	5.35	9.90	5.76	AD < MA*, EA*
AWARENESS	7.70	3.72	6.70	3.34	8.00	3.40	8.50	4.12	ND
CLARITY	4.05	2.68	3.85	2.58	4.80	4.16	4.70	4.25	ND

AD, adults; LA, late adolescents; MA, middle adolescents; EA, early teens; NONACCEPTANCE, nonacceptance of emotional response; GOALS, difficulties engaging in goal directed behavior; AWARENESS, lack of emotional awareness; CLARITY, lack of emotional clarity. M, mean; SD, standard deviation, *p < 0.05; ND, no difference.

### 3.2 Cooperative responses

Regarding the second question, we observed differences between groups [*F*(3) = 24.617; *p* < 0.001, η^2^ = 0.21] with higher number of cooperation responses in Adults compared to Middle Adolescents (*p* < 0.05), and Early Adolescents groups (*p* < 0.001), but not with Late Adolescents group (*p* = 0.415). Likewise, we did not observe differences between the other adolescent groups: Late Adolescents and Middle Adolescents (*p* = 1.000); Late Adolescents and Early Adolescents (*p* = 0.075), Middle Adolescents and Early Adolescents (*p* = 1.000). In the same direction, we observed differences between groups [*F*(3) = 24.617; *p* < 0.001, η^2^ = 0.21] with lower numbers of defecting responses in Adults compared to Middle Adolescents (*p* < 0.05), and Early Adolescents groups (*p* < 0.001).

### 3.3 General correlations

We observed a positive correlation between the Perspective Taking sub-scale of IRI and cooperation responses score (*r* = 0.30, *p* < 0.01), and a negative correlation between the Perspective Taking sub-scale and defect responses score (*r* = −0.30, *p* = 0.006). Regarding emotional regulation scale, we observed a positive correlation between the Difficulties engaging in goal-directed behavior (*r* = 0.33, *p* < 0.05) and Lack of emotional awareness (*r* = 0.40, *p* < 0.05) sub-scales of DERS with Personal Distress sub-scale of IRI.

## 4 Discussion

This study aimed to identify: (1) the differences in the levels of empathy and emotional regulation during the different stages of adolescence and, (2) their relationship with cooperative behavior in the prisoner’s dilemma. Firstly, the results showed that the three older groups (Adults, Late Adolescents, and Middle Adolescents) were distinguished from the younger group (Early Adolescents), and they showed higher levels of Perspective Taking (cognitive empathy) and lower levels of Personal Distress (affective empathy). Secondly, in terms of emotional regulation, Early Adolescents participants presented higher levels than the other groups, i.e., more Difficulties in engaging goal-directed behaviors (GOALS factor). The presence of both findings could influence the choice of empathetic-type cooperative behavior that tends to emerge at late adolescence. Thus, the present study brings some evidence to distinguish between the different stages of adolescence and adulthood based on the levels of empathy, emotional regulation, and cooperative behaviors.

### 4.1 Empathy and emotional regulation level’s during the different stages of adolescence and adulthood

We distinguished older groups from both younger groups, by higher scores in the cognitive empathy component Perspective Taking. This suggests that older participants have a better ability to understand and adapt to circumstances based on the perspective and point of view of the other younger participants (Davis, 1980; Filippetti et al., 2012; Christov-Moorea et al., 2014). As a result, it appears that Perspective Taking let them show cooperative behavior based on empathic responses. In addition, some authors have related Perspective Taking to the term theory of mind, which implies the ability to understand and predict the behavior of another person, their knowledge, intentions, and beliefs (Tirapu-Ustárroz et al., 2007). Moreover, Perspective Taking has linked with the development of the cerebral cortex and personal experience because the empathic response bases on what is observed, verbal information, and information accessible from memory (Mestre Escrivá et al., 2002). However, several studies that report different levels of Perspective Taking in the adult population support the fact that this skill does not depend exclusively on age (Eisenberg et al., 2006). Other factors that have been related to the acquisition and functioning of Perspective Taking during adolescence are: the influence of the attachment style (Paez and Rovella, 2019), sympathy (Carlo et al., 2007), failures in emotional regulation (Moreno Bataller et al., 2019), among others (see Eisenberg et al., 2006). It would be interesting to investigate how these variables relate to empathetic responses in future studies. Another important component of empathy between groups was the Personal Distress level. The argument in favor of the Early Adolescents group is that they could show higher levels of anxiety and distress when observing another person in the face of negative experience. Besides, our results are consistent with previous studies that have found similar results, in which Personal Distress decreases with age (Eisenberg et al., 2005). Evidence from other studies has indicated that brain areas related to affective empathy, which has been related to emotional contagion and mirror neurons (López et al., 2014), mature more quickly than those for cognitive empathy (Filippetti et al., 2012; Gómez, 2016; Zavala et al., 2018).

Regarding emotional regulation skills, interestingly we found that the Early Adolescents group obtained higher scores in the sub-scale Difficulties engaging in goal-directed behavior of DERS, which suggests that the Early Adolescents group shows low control when negative emotions interfere with effective actions to achieve their goals (Muñoz Martínez et al., 2016). On the contrary, higher development of regulatory ability makes it possible to better modulate the aversive emotion generated by personal discomfort. In same vein, Eisenberg et al. (2005) reported similar results by assessing changes in prosocial responses in adolescence (Eisenberg et al., 2005); furthermore, same authors noted that, during early adolescence, emotional reactivity strongly increases and adolescents have difficulties to modulate their empathic affect. Thus, when early adolescents experience negative emotion, they will not be able to regulate their emotions effectively (Eisenberg et al., 2005).

### 4.2 Cooperative behavior, empathy and emotional regulation during the different stages of adolescence and adulthood

Regarding the cooperative behaviors evaluated in the prisoner’s dilemma, the Adults group showed higher cooperative responses than Middle Adolescents and Early Adolescents groups, but not with the Late Adolescents group. This kind of response is consistent with previous results (Belli et al., 2012). According to the authors, the results could be due to the development of cognitive skills, such as the ability to plan, working memory, monitoring, organization, cognitive flexibility and semantic categorization, which are found in adults aged 19–35 years. In other words, we can conceive of them being largely due to the development of executive functions. Evidence from this study showed that cooperative responses could also be associated to the level of Perspective Taking, in which Adults and Late Adolescents groups showed higher levels than the other two younger groups. Furthermore, the correlations between Perspective Taking and cooperative responses are in direction of this proposition. Perspective Taking implies the ability to consider a situation from different points of view, which makes it easier to make predictions regarding the behavior of others (López et al., 2014). The higher cooperation responses from Adults and Late Adolescents groups suggest that they chose to adopt the other’s perspective and make choices that would increase the chance of achieving the goal of the game by predicting the other participant’s next choice.

On the other hand, the choices from Middle Adolescents and Early Adolescents groups in the paradigm seem to be more associated to their Personal Distress component. Early Adolescents group showed higher levels than the Middle Adolescents and Late Adolescents groups in this variable. In addition, we found a positive correlation between Difficulties engaging in goal-directed behavior and Lack of emotional awareness sub-scale of DERS and the Personal Distress sub-scale of IRI. Both variables of DERS refer to difficulties in reaching a goal due to emotional interference (Muñoz Martínez et al., 2016). These data suggest that the cooperation choices of the Middle Adolescents group were influenced by Personal Distress and not by Perspective Taking. This let us deduce that the cooperative choices that they presented were not induced by empathetic behavior, but by avoiding emotional aversion. Thus, the feelings of anxiety and discomfort that the participants could present during the test led them to try to relieve their aversive state and select the option of cooperating with the other participant (Carrasco Ortiz et al., 2011; López et al., 2014).

Additionally, a recent study reported that adolescents show lower cooperative behaviors when they are under time–pressure than when they have time to reflect which suggest they are intuitively selfish (Nava et al., 2023). Nevertheless the sample of study was age range 12–19 years old and the authors did not distinguish between adolescent’s periods. Interestingly, in the present study we find that Late Adolescents did not show differences in cooperative behaviors with Adult and younger groups neither. A likely explanation is that the participants were not under time–pressure, so they could show similar cooperation. However, they are different in empathy and emotional regulation in comparison to younger groups, which suggests a different motivation although the behavior is the same. It would be interesting to prove in future studies whether under time–pressure each group of adolescent’s period shows cooperative behavior according their empathic characteristics (Personal Distress/Perspective Taking) and emotional regulation level (Difficulties engaging in goal-directed behavior).

### 4.3 Study limitations

Some limitations of our study lie in the fact that we only evaluated males, so it would be convenient to corroborate the results with women. We selected only males because it was not possible to balance the same number of participants of women because difficulties to contact participants during the pandemic period COVID-19. In addition, although the size of sample were 80 participants, it seems advisable to increase the current number of participants for each group (*n* = 20) in future studies. Similarly, we could not verify if the participants suspected at any time that their partner was not a real person and responses depended on computer programming. It would be convenient in future studies to consider these variables to reaffirm and extend the results reported here.

## 5 Conclusion

The results of this study bring original data that the level of empathy and emotional regulation during the different stages of adolescence could be related to cooperative behavior in the face of paradigms such as the prisoner’s dilemma. Particularly, the results suggest that Perspective Taking component is reached approximately at the beginning of Late Adolescence, and the cognitive development linked with Difficulties engaging in goal-directed behavior (emotional regulation) shows a similar level to the Adults from Middle Adolescence. According to the findings, the presence of both factors seems to be consistent with the idea that they contribute to the progressive inhibition of personal discomfort (Personal Distress), which is quite prevalent during Early Adolescence. This suggests that the gradual installation of such inhibition could be a determining factor to favor a higher amount of cooperative responses from Middle Adolescence. Present findings also suggest that, at the beginning of adolescence, cooperative behavior occurs mostly to social reinforcement rather than empathy towards others while during late adolescence, when the cognitive skills as Perspective Taking and Emotional Regulation are developed, the cooperative behavior is carried out by understanding the others and empathy. In this context, it would be interesting that future studies could evaluate whether social reinforcement of cooperative behaviors facilitates the development of the cognitive skills or whether these arise independently due to brain maturity. Finally, in a forward-looking approach, our results raise the question of new educational strategies that could capitalize on the early role of social reinforcement in the emergence of cooperative behaviors and their impact on cognitive acquisitions and well-being of adolescents.

## Data availability statement

The original contributions presented in the study are publicly available. This data can be found here: https://osf.io/v6jcn/?view_only=9bec409e4af042d8b1157c17f3ea91ad.

## Ethics statement

The studies involving humans were approved by the research and ethics committee of the Department of Psychology of Benemérita Universidad Autónoma de Puebla (SIEP140/2021). The studies were conducted in accordance with the local legislation and institutional requirements. Written informed consent for participation in this study was provided by the participants’ legal guardians/next of kin.

## Author contributions

EM-V: Conceptualization, Data curation, Formal analysis, Investigation, Methodology, Resources, Supervision, Validation, Visualization, Writing – original draft, Writing – review & editing. SP-J: Data curation, Formal analysis, Funding acquisition, Investigation, Methodology, Project administration, Resources, Software, Visualization, Writing – original draft. AD: Data curation, Formal analysis, Writing – review & editing. HS: Formal analysis, Resources, Supervision, Validation, Visualization, Writing – review & editing.
